# Three-Dimensional Wind Measurement Based on Ultrasonic Sensor Array and Multiple Signal Classification

**DOI:** 10.3390/s20020523

**Published:** 2020-01-17

**Authors:** Bian Ma, Jing Teng, Huixian Zhu, Rong Zhou, Yun Ju, Shi Liu

**Affiliations:** School of Control and Computer Engineering, North China Electric Power University, Beijing 102206, China; bian_MA@ncepu.edu.cn (B.M.); zhuhuixian@ncepu.edu.cn (H.Z.); zhourong@ncepu.edu.cn (R.Z.); juyun1982@ncepu.edu.cn (Y.J.); liushi@ncepu.edu.cn (S.L.)

**Keywords:** wind measurement, ultrasonic sensor array, 3D vector, MUSIC algorithm

## Abstract

The wind power industry continues to experience rapid growth worldwide. However, the fluctuations in wind speed and direction complicate the wind turbine control process and hinder the integration of wind power into the electrical grid. To maximize wind utilization, we propose to precisely measure the wind in a three-dimensional (3D) space, thus facilitating the process of wind turbine control. Natural wind is regarded as a 3D vector, whose direction and magnitude correspond to the wind’s direction and speed. A semi-conical ultrasonic sensor array is proposed to simultaneously measure the wind speed and direction in a 3D space. As the ultrasonic signal transmitted between the sensors is influenced by the wind and environment noise, a Multiple Signal Classification algorithm is adopted to estimate the wind information from the received signal. The estimate’s accuracy is evaluated in terms of root mean square error and mean absolute error. The robustness of the proposed method is evaluated by the type A evaluation of standard uncertainty under a varying signal-to-noise ratio. Simulation results validate the accuracy and anti-noise performance of the proposed method, whose estimated wind speed and direction errors converge to zero when the SNR is over 15 dB.

## 1. Introduction

As wind energy is considered one of the most promising renewable energies, it is widely used for electric power generation [1,2]. However, despite the continuing increase in installed capacity of wind power worldwide, the wind power that is actually utilized is much less than would be expected, because of the unstable, intermittent, and highly volatile nature of wind. Furthermore, due to the characteristics of randomness and fluctuation, the electricity generated by the wind will be excessive or insufficient, making it difficult to integrate into the power grid [3,4]. On one hand, one possibility aiming at the redundant electricity is to simply abandon the fluctuating wind power [5,6]. However, that would come with not only a huge loss of electricity but also a mass waste of wind resources and wind power equipment. According to a report from the National Energy Administration of China [7], the national average rate of wind abandonment in 2018 is ~7%, which is equivalent to 27.7 billion kWh, resulting in an enormous loss of about US $2.1 billion. On the other hand, a solution for overcoming the deficiency of wind power generation is to store the wind energy. Hybrid wind/compressed air energy storage (CAES) systems are used to transform the intermittent wind resources into a constantly available power supply in Germany, the USA, and even remote Arctic areas [8]. However, the underground geology may bring risks to practical applications of CAES [9]. In contrast, accurate wind measurement in a 3D space can provide data that can be used to control wind turbines, which further maximizes wind utilization efficiency [10]. In addition, wind measurement can be used to estimate the corresponding power generation, thus contributing to the system scheduling and energy dispatching, and, ultimately, the integration of wind power in the grid [11]. In summation, precise wind speed and direction measurements in a 3D space are of vital importance to wind energy utilization and the wind power industry.

Light detection and ranging (LIDAR) technology is one of the most popular wind measurements [12]. However, LIDAR can only measure the wind speed component along the line of sight, a disadvantage known as “the Cyclops dilemma” [13,14]. Therefore, the wind distribution measurement in a 3D space would require the very costly installment and deployment of several LIDARs in the wind farm. In contrast, wind measurement sensors are much cheaper and could be deployed around the wind field in distances of miles to provide information for previewing wind measurement. Several types of wind measurement sensors have been employed by researchers, including cup anemometers [15,16,17], thermal anemometers [18,19,20], and ultrasonic anemometers [21,22,23]. However, measurements from cup anemometers often suffer from errors caused by wear and tear on the internal rotating bearings, and frequent inspection or calibration is required to ensure measurement accuracy [24,25]. The performance of cup anemometers can also be affected by the measuring environment. For example, cup anemometers are prone to jamming in humid environments, since water vapor can penetrate their bearings [26]. Thermal anemometers also have difficulties coping with harsh environments. In addition, their sensitivity to changes in the velocity field decreases as the wind speed increases, which results in the limited application of thermal anemometers [27]. Unlike cup and thermal anemometers, ultrasonic anemometers incur relatively little maintenance cost due to the absence of mechanical rotating parts [28]. Furthermore, ultrasonic anemometers have high sensitivity and can perform well in harsh environments [29]. Finally, the high precision and wide measuring range of ultrasonic anemometers make them the first choice for wind measurement [30].

The main wind measurement methods used in ultrasonic sensors are the vortex [31,32] and the time-of-flight (TOF) [29,33,34]. The vortex method determines wind speed by measuring the frequency of the vortex, based on the proportional relationship between the average flow velocity and the eddy current frequency. However, measuring the wind direction using the vortex method is complex [33,34]. The TOF method is easier to implement, and can obtain both the wind speed and direction simultaneously. The wind speed is calculated by the transit time of ultrasonic signals between the transmitter and receiver, whereas the wind direction can be determined according to the obtained speed components and positional relationship between the sensors [33]. As a result, the accuracy of wind measurement thoroughly depends on the obtained transmission time, which is difficult to measure precisely, particularly in the case of low signal-to-noise ratio (SNR). To overcome the limitations of the TOF method, Li et al [35] proposed a novel wind measurement method. The method combined the multiple signal classification (MUSIC) algorithm proposed by R. O. Schmidt [36] and an arc ultrasonic sensor array to measure the wind speed and horizontal direction in the 2D space. The MUSIC algorithm is a typical representative of array signal processing, which transforms the time measuring problem to spectrum searching, and it employs spectrum search to estimate the direction of arrival (DOA) of the array signal. Due to the transformation, the MUSIC algorithm can effectively suppress the noise existing in complex environments [35]. Therefore, the measurement proposed by Li et al in [35] can precisely measure the wind in a 2D space even in a low SNR case. 

Although the method in [35] can measure the wind speed and horizontal direction in the 2D space, it fails to consider the vertical wind direction information. The vertical direction has a significant influence on certain industrial applications. For example, by studying the performance of a small vertical axis wind turbine, Lee et al found that the performance of the generator is significantly improved when the vertical wind direction is less than or equal to 45°, generating more than 90% of the power [37]. This is undoubtedly an important discovery for the wind power industry. To fully utilize the natural wind in the 3D space, we measure the wind as a 3D vector, whose direction and magnitude correspond to the direction and speed of the wind. In addition, the wind direction is decomposed into horizontal and vertical components, namely, the azimuth and pitch angles, respectively. We adopted the MUSIC algorithm for wind measurement due to two advantages: the first is that the MUSIC algorithm makes the measurement accuracy independent of the precision of the signal transmission time in the wind. In addition, the environment noise and the sampling noise are suppressed, which reduces the influence of noise on the accuracy of measurement results [35]. The second advantage is that the MUSIC algorithm can be combined with a variety of sensor arrays and applied to different scenarios. Except for the arc array mentioned by Li et al. [35], the MUSIC algorithm can also be used on a uniform circular array [38], which estimates the dual angle of azimuth and pitch angle of the incident signal. In addition, other array structures, such as the uniform linear (ULA) [39], L-shaped array [40], and conformal array [41], can be combined with the MUSIC algorithm for diverse scenarios and purposes. To measure these components simultaneously, especially adding the measurement of the pitch angle, we propose a novel semi-conical ultrasonic sensor array in 3D space, which is different from the planar array proposed by Li et al. [35]. The array consists of six ultrasonic sensors, where the transmitting sensor is on the peak of the cone, and the five receiving sensors are evenly positioned on the undersurface arc of the cone. According to the spatial relationship between the transmitter and the receiver, we simulate a 3D wind vector, and calculate the theoretic transmission time of ultrasonic signal. The theoretic transmission time is then used to determine the array manifold matrix, which is critical for the implementation of the MUSIC algorithm. After receiving the true ultrasonic signal, the MUSIC algorithm divides the covariance matrix of the received information into signal and noise subspaces. Under ideal conditions, the signal and noise subspaces are completely orthogonal, and can be used to calculate the spectral function. By searching for the peak value of the spectrum with the speed searching step of 0.1 m/s and the direction searching step of 1º, we can obtain the corresponding information of wind speed, pitch angle, and azimuth angle simultaneously. Simulation results indicate that the proposed method can measure the azimuth angle in (0°, 360°), the pitch angle in (0°, 90°). Furthermore, the proposed method demonstrates better performances than the state-of-the-art method in terms of convergence speed, estimation error, and variance.

The rest of the study is organized as follows. Section 2 describes the structure of the proposed sensor array and the wind measurement principle. Section 3 discusses the simulations and a comparison with the state-of-the-art method, which analyzes the accuracy and anti-noise performances of the methods. Section 4 includes our conclusions and suggestions for further research.

## 2. 3D Wind Measurement

### 2.1. The Ultrasonic Sensor Array

**Figure 1:**
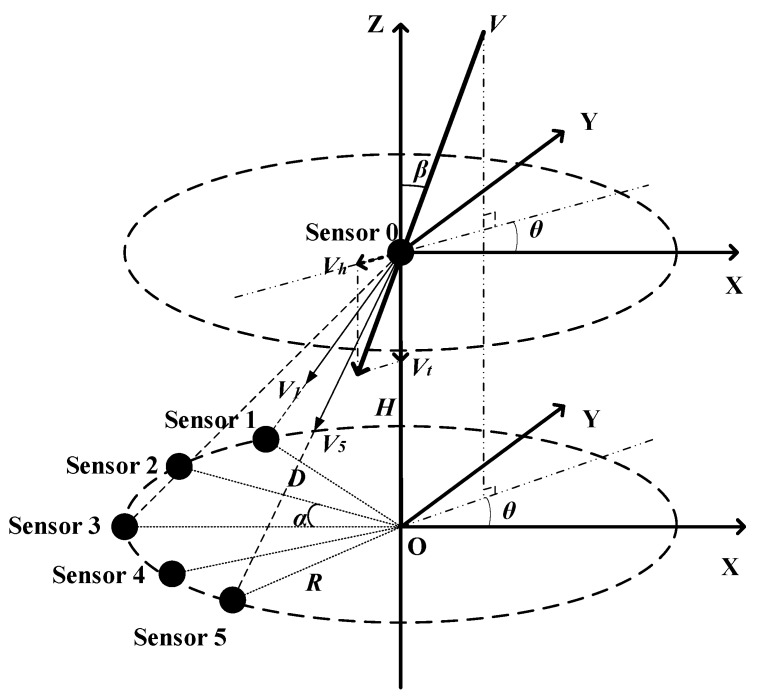
Structure of the ultrasonic sensor array in the 3D space.

**Figure 2:**
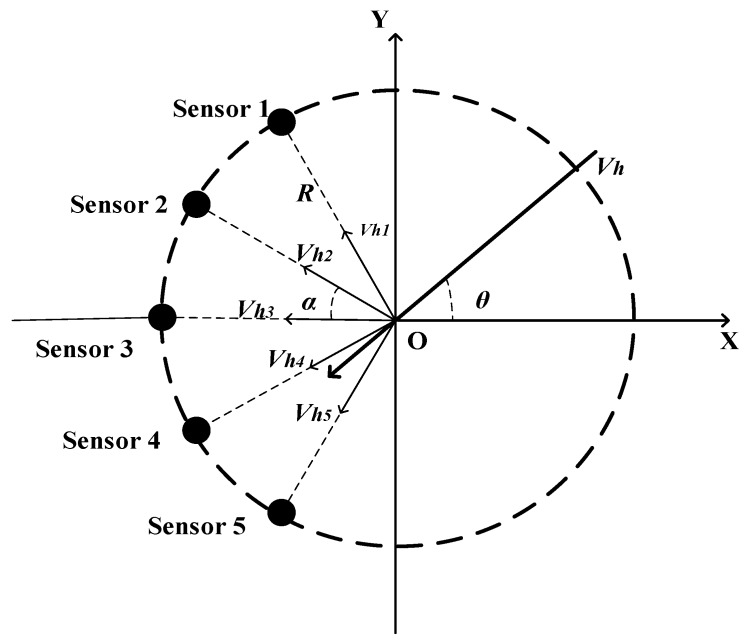
Structure of the receiving ultrasonic sensors on the horizontal plane.

Wind measurements can be based on several types of ultrasonic sensor array structures, of which the arc ultrasonic sensor array and the ULA structure are the most prominent [35,39]. When combined with the MUSIC algorithm, the arc array structure takes precedence over the ULA structure in terms of estimate variance [39]. However, these experiments were performed in the 2D space. To measure the natural wind in the 3D space, we propose a novel semi-conical structure sensor array based on the arc array structure. The sensor array consists of six ultrasonic sensors, numbered 0–5, wherein Sensor 0 is used for transmitting ultrasonic signals, and Sensors 1–5 are receiving sensors. Figure 1 depicts the semi-conical structure in the 3D space. The transmitting ultrasonic sensor (Sensor 0) is located on the vertex of the cone, with height H. The five receiving ultrasonic sensors (Sensors 1–5) are evenly positioned on the undersurface arc of the cone. To clearly display the positional relationship of the five receiving sensors on the horizontal plane, we plot the undersurface of the cone in Figure 2. As illustrated in Figure 1 and Figure 2, the five receiving sensors are evenly located on the semi arc with the center O and the radius R, where the angle between every two adjacent receiving sensors is α. The distance D between the transmitting sensor and each receiving sensor can be deduced by $D=\sqrt{R^{2}+H^{2}}$.

### 2.2. Measuring Principle

#### 2.2.1. Premise Assumptions

The transmission of signal through wireless channel is complicated, which makes it difficult to completely describe the physical environment and establish a rigorous mathematical model. As mentioned in the literature [35], the arrival signals of the wavefront in the array system can be considered a spherical wave. Therefore, the propagation time of the signal to each sensor is not only related to the direction, but also to the distance of the signal travels. In this case, the wind speed and wind direction information can be estimated with the MUSIC algorithm on the grounds that the output data matrix of the sensor array is only related to the wind speed and wind direction.

Although the wind speed and direction can be regarded as continuous signals, we discretize them by the sampling frequency of 100 MHz, resulting in the short sampling period of 10 ns. According to the validity of Taylor’s hypothesis of frozen turbulence [42], we assume that the amplitude of the wind speed and direction are approximately constant throughout a sampling period. The propagation speed of the signal on each path is only affected by the component of the wind vector parallel to the propagation path as in transit-time Ultrasonic Flowmeter [43,44]. The key to adopting the MUSIC algorithm is to build the array manifold matrix, which implies the information of wind speed and direction. Ultimately, the measured wind information can be regarded as the average ones over the sampling period.

#### 2.2.2. Signal Model

In general, when M narrowband signals are incident on an array containing N array elements, the received signal can be expressed by the following complex envelope form,

(1) $$\{\begin{array}{c} s_{i}\left(t\right)=u_{i}\left(t\right)e^{j\left(\omega_{0}t+\varphi \left(t\right)\right)}\\ s_{i}\left(t-\tau \right)=u_{i}\left(t-\tau \right)e^{j\left(\omega_{0}\left(t-\tau \right)+\varphi \left(t-\tau \right)\right)} \end{array},\;i=1,2,\cdots ,M,$$

where $u_{i}\left(t\right)$ represents the amplitude of the signal. φ(t) denotes the phase of signal. $\omega_{0}$ is the angular frequency of signal and $\omega_{0}=2\pi f$, where f is the frequency of signal.

According to the narrowband assumption, we can obtain:$u_{i}\left(t-\tau \right)\approx u_{i}\left(t\right)$ and $u_{i}\left(t-\tau \right)\approx u_{i}\left(t\right)$, where i=1,2,⋯,M [45]. Combined with Equation (1), it can be derived that

(2) $$s_{i}\left(t-\tau \right)\approx s_{i}\left(t\right)e^{-j2\pi f\tau }.$$

Therefore, it can be obtained that the expression of all signals received by the $k_{th}$ array element in the array is as follows; $x_{k}\left(t\right)=\sum_{i=1}^{M}g_{ki}s_{i}\left(t-\tau_{ki}\right)+n_{k}\left(t\right),k=1,2,\ldots ,N$, where $n_{k}\left(t\right)$ is the noise of $k_{th}$ array element at time t, $\tau_{ki}$ denotes the transmission time when the $i_{th}$ signal reaches the $k_{th}$ array element, and $g_{ki}$ is the gain of $i_{th}$ signal on the $k_{th}$ array element. Based on the assumption that all array elements are isotropic and the coupling between them can be negligible, the $g_{ki}$ can be averaged to 1. Combined with Equation (2), the signal received by $k_{th}$ array element can be expressed as follows: $x_{k}\left(t\right)=\sum_{i=1}^{M}s_{i}\left(t\right)e^{-j2\pi f\tau_{ki}}+n_{k}\left(t\right),k=1,2,\ldots ,N$.

Because there are five receiving sensors and only one signal resource in this paper, that is, N=5,M=1, we use S(t)=s(t) to denote the original signal transmitted by the Sensor 0. The received signal $x_{k}\left(t\right)$ at the $k_{th}$ sensor can be expressed as follows,

(3) $$x_{k}\left(t\right)=S\left(t\right)*e^{-j2\pi f\tau_{k}}+n_{k}\left(t\right),\;$$

where k=1,2,⋯5, and $\tau_{k}$ denotes the transmitting time. $n_{k}\left(t\right)$ denotes the noise on the $k_{th}$ receiving sensor, which is assumed to be an independently and identically distributed (i.i.d.) AWGN.

To speed up signal processing, we vectorize the received signal as follows.

(4) $$\left(\begin{array}{c} x_{1}(t)\\ x_{2}(t)\\ x_{3}(t)\\ x_{4}(t)\\ x_{5}(t) \end{array}\right)=\left(\begin{array}{c} e^{-j2\pi f\tau_{1}}\\ e^{-j2\pi f\tau_{2}}\\ e^{-j2\pi f\tau_{3}}\\ e^{-j2\pi f\tau_{4}}\\ e^{-j2\pi f\tau_{5}} \end{array}\right)S(t)+\left(\begin{array}{c} n_{1}(t)\\ n_{2}(t)\\ n_{3}(t)\\ n_{4}(t)\\ n_{5}(t) \end{array}\right)$$

Equation (4) can be further rewritten in the matrix form:

(5) X(t)=A·S(t)+N(t),

where N(t) is the noise matrix and A is the array manifold matrix described by Equation (6):

(6) $$A={\left[e^{-j2\pi f\tau_{1}},e^{-j2\pi f\tau_{2}},e^{-j2\pi f\tau_{3}},e^{-j2\pi f\tau_{4}},e^{-j2\pi f\tau_{5}}\right]}^{T}.$$

It is obvious that the key to obtaining array manifold matrix A is to obtain the transmission time of the ultrasonic signal to each receiving sensor. The transmission time can be calculated by analyzing the structure of the sensor array and the influence of natural wind on the signal propagation. As a result, the wind measurement problem is transformed into an identification problem of wind speed V, azimuth angle θ, and pitch angle β from the array manifold matrix A. The influence of the natural wind on the ultrasonic signal is thus reduced to the signal transmitting time τ in matrix A. In the case of no wind, τ should be identical at all five receiving sensors, which is

(7) $$\tau =\frac{D}{c}=\frac{\sqrt{R^{2}+H^{2}}}{c},$$

where c=340 m/s is the propagation speed of ultrasonic wave. When the transmission of the ultrasonic signal is influenced by the wind, $\tau_{i}$ would be different for each receiving sensor, resulting in a more complex calculation. To simplify the calculation, we decompose the natural wind speed V into a vertical wind speed $V_{t}$ and a horizontal wind speed $V_{h}$ by vector decomposition, accordingly

(8) $$\begin{array}{c} V_{h}=V\sin\beta \\ V_{t}=V\cos\beta \end{array}.$$

Similarly, the original propagation speed c of ultrasonic wave is decomposed into $c_{h}$ and $c_{t}$, which are as follows,

(9) $$\begin{array}{c} c_{h}=c\frac{R}{D}\\ c_{t}=c\frac{H}{D} \end{array}.$$

However, under the influence of wind, the actual speed of the ultrasonic signal is changed. We use $V_{i}$ to denote the ultrasonic signal speed received at the $i_{th}$ sensor. The value of $V_{i}$ is calculated based on the principle of vector decomposition and the positional relationship of the sensors in the 3D space. Accordingly, the horizontal components of $V_{i}$ are as follows,

(10) $$\begin{array}{c} V_{h1}=V_{h}\cos\left(\theta +2\alpha \right)+c_{h}\\ \begin{array}{c} V_{h2}=V_{h}\cos\left(\theta +\alpha \right)+c_{h}\\ V_{h3}=V_{h}\cos\theta +c_{h}\\ V_{h4}=V_{h}\cos\left(\theta -\alpha \right)+c_{h}\\ V_{h5}=V_{h}\cos\left(\theta -2\alpha \right)+c_{h} \end{array} \end{array}.$$

The vertical components $V_{ti}$ are identical for all the receiving sensors, which can be calculated by

(11) $$V_{ti}=V_{t}+c_{t}.$$

Therefore, we can infer $V_{i}$ by synthesizing $V_{hi}$ and $V_{ti}$ as follows,

(12) $$V_{i}=\sqrt{{V_{hi}}^{2}+{V_{ti}}^{2}}\cos\left|\tan^{-1}\left(\frac{V_{hi}}{V_{ti}}\right)-\tan^{-1}\left(\frac{R}{H}\right)\right|.$$

Accordingly, the transmission time $\tau_{i}$ of the signal at the $i_{th}$ receiving sensor can be represented as follows,

(13) $$\tau_{i}=\frac{D}{V_{i}},\;i=1,2,\cdots 5.$$

By combining Equations (8)–(13), we can obtain the relationship between the wind and the transmission time τ of the ultrasonic signal. However, it is not easy to retrieve the wind information V, θ, and β directly from the transmission time. Therefore, the MUSIC algorithm is used to identify the wind speed V, azimuth angle θ, and pitch angle β, as implied in the matrix.

### 2.3. Wind Measurement Based on MUSIC Algorithm

The process of the MUSIC algorithm is to first calculate the covariance matrix $R_{X}$ of the received signal X(t); next, perform eigenvalue decomposition on the obtained covariance matrix $R_{X}$, resulting in a signal subspace $U_{S}$ and noise subspace $U_{N}$; and finally, based on the orthogonality of the array manifold matrix A and the noise subspace $U_{N}$, the corresponding V, θ, and β can be obtained by performing spectral peak searching.

Specifically, the covariance matrix $R_{X}$ of the signal matrix X(t) can be obtained by Equation (14):

(14) $$\begin{array}{ll} R_{X} & =E\left[X\left(t\right)X^{H}\left(t\right)\right]\\ & =E\left[{\left(A\cdot S\left(t\right)+N\left(t\right)\right)\left(A\cdot S\left(t\right)+N\left(t\right)\right)}^{H}\right]\\ & =AE\left[S\left(t\right)S^{H}\left(t\right)\right]A^{H}+E\left[N\left(t\right)N^{H}\left(t\right)\right]\\ & =AR_{S}A^{H}+R_{N}\\ & =AR_{S}A^{H}+\sigma^{2}I, \end{array}$$

where ^H^ denotes the conjugate transpose. $R_{S}$ and $R_{N}$ are the correlation matrices of signal and noise, respectively. $\sigma^{2}$ is the variance of noise, and I is the unit matrix.

As $R_{X}$ is symmetric, we can conduct eigenvalue decomposition on it, resulting in

(15) $$R_{X}=U\Sigma U^{H}=U_{S}\Sigma_{S}U_{S}^{H}+U_{N}\Sigma_{N}U_{N}^{H},$$

where $U=\left[U_{S},U_{N}\right]$, $\Sigma =\left[\begin{array}{cc} \Sigma_{S} & \\ & \Sigma_{N} \end{array}\right]=\left[\begin{array}{cccc} \lambda_{1} & & & \\ & \lambda_{2} & & \\ & & \ddots & \\ & & & \lambda_{N} \end{array}\right]$, where $\lambda_{1}\geq \lambda_{2}\geq \cdots \geq \lambda_{M}>\lambda_{M+1}=\cdots =\lambda_{N}=\sigma^{2}$. Therefore, $\sigma^{2}$ is the smallest eigenvalue of $R_{X}$, which corresponds to N−M eigenvectors. These N−M eigenvectors comprise the noise subspace $U_{N}$. The other M eigenvalues are relevant to the signals, whose eigenvectors constitute $U_{S}$, the signal subspace. $R_{X}$ is a Hermitian matrix, thus the signal subspace $U_{S}$ and noise subspace $U_{N}$ are orthogonal, namely, $U_{S}^{H}U_{N}=0$. We can further infer that $R_{X}U_{N}=\left[U_{S},U_{N}\right]\Sigma \left[\begin{array}{c} U_{S}^{H}\\ U_{N}^{H} \end{array}\right]U_{N}=\left[U_{S},U_{N}\right]\left[\begin{array}{cc} \Sigma_{S} & \\ & \Sigma_{N} \end{array}\right]\left[\begin{array}{c} 0\\ I \end{array}\right]=\sigma^{2}IU_{N}$. However, referring to Equation (14), we can also get $R_{X}U_{N}=AR_{S}A^{H}U_{N}+\sigma^{2}IU_{N}$. Therefore, $AR_{S}A^{H}U_{N}=0$. We left multiply the equation by $U_{N}^{H}$, resulting to $U_{N}^{H}AR_{S}A^{H}U_{N}={\left(A^{H}U_{N}\right)}^{H}R_{S}A^{H}U_{N}=0$. Since $A\neq 0,{\;R}_{S}\neq 0$, we can derive that $A^{H}U_{N}=0$.

In practical applications, $R_{X}$ cannot be directly obtained, while only the sampled covariance $\hat{R_{X}}$ can be calculated by

(16) $$\hat{R_{X}}=\frac{1}{L}\overset{L}{\underset{i=1}{\sum }}X\left(t\right)X{\left(t\right)}^{H},$$

where L is the number of samplings. Theoretically, when  L→∞, $\hat{R_{X}}$ and $R_{X}$ are consistent. In our experiment, we set L=1000 to provide the best possible approximation of $R_{X}$.

Similarly, we can also perform eigenvalue decomposition on$\;\hat{R_{X}}$:

(17) $$\hat{R_{X}}=U_{\hat{S}}\Sigma_{\hat{S}}U_{\hat{S}}^{H}+U_{\hat{N}}\Sigma_{\hat{N}}U_{\hat{N}}^{H},$$

where $U_{\hat{S}}$ indicates the signal subspace consisting of the signal eigenvectors and $U_{\hat{N}}$ represents the noise subspace. However, due to the deviation between $\hat{R_{X}}$ and${\;R}_{X},\;A^{H}U_{\hat{N}}\neq 0$. To retrieve the wind information V, θ, and β from the matrix A, the MUSIC algorithm constructs a spectral estimation formula:

(18) $$P_{music}=\frac{1}{A_{\left(V,\;\theta ,\;\beta \right)}^{Η}U_{\hat{N}}U_{\hat{N}}^{Η}A_{\left(V,\;\theta ,\;\beta \right)}}.\;$$

The maximum value of$\;P_{music}$, namely the spectrum peak, corresponds to the smallest value of $A^{H}U_{\hat{N}}$. According to Equations (4), (6), and (18), we calculate the theoretical maximum value of $P_{music}$ by searching through the measuring range of V, θ, and β. The corresponding values of  V, θ, and β that generate the spectral peak are the measured results of the wind speed and direction information. Therefore, the wind speed V, azimuth angle θ, and pitch angle β of the natural wind in the 3D space are simultaneously obtained.

## 3. Simulations and Results

### 3.1. Simulations

Simulations are conducted using the ultrasonic sensor array shown in Figure 1, where the radius R and the height H are both set to 1 m, and the angle α between every two adjacent receiving sensors is 30°. The propagation speed c and frequency f of the transmitted ultrasonic signal are 340 m/s and 40 kHz, respectively. The snapshot number L is 1000. With respect to the spectral search, we set the azimuth angle searching range to (0, 360°), the pitch angle range to (0, 90°), and the searching step length to 1°. The searching range of the wind speed is (0, 60) m/s with the searching step length of 0.1 m/s for the MUSIC algorithm.

**Figure 3:**
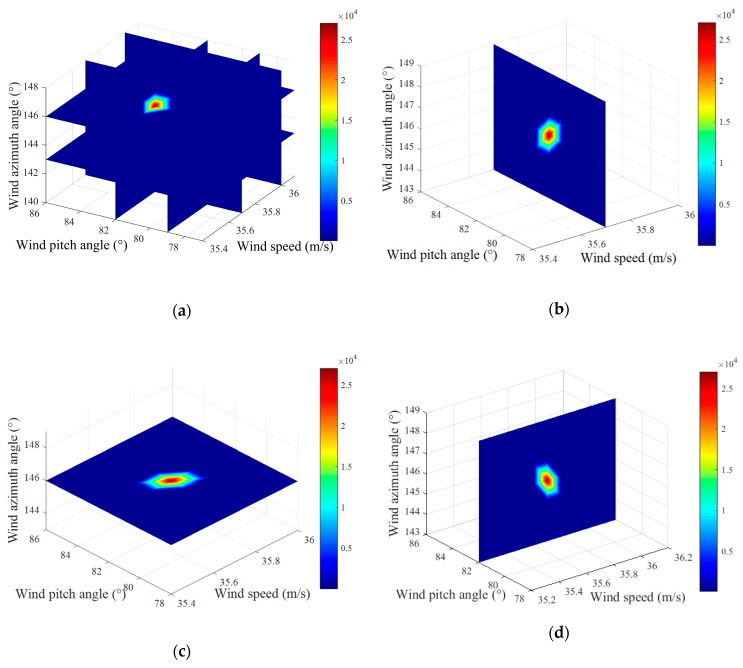
Result of the spectral peak searching: (**a**) result of peak searching, (**b**) the slice result in the direction of wind speed, (**c**) the slice result in the direction of wind azimuth angle, and (**d**) the slice result in the direction of wind pitch angle.

Based on the above experimental parameters, we performed the simulations. The noise on the receiving sensor was simulated by an AWGN with zero-mean, whose variance is the average power of noise. Figure 3 depicts the spectral searching result of SNR at 20 dB. As illustrated in Figure 3, the darkest red color is concentrated at the position corresponding to the peak value of the spectral function. In this case, the wind speed V= 35.7 m/s, wind azimuth angle θ= 146°, and wind pitch angle β= 82°. Figure 3b–d shows the slice results in the direction of wind speed, wind azimuth angle, and wind pitch angle, which demonstrate the estimation results of these components. These results are consistent with those in Figure 3a.

**Figure 4:**
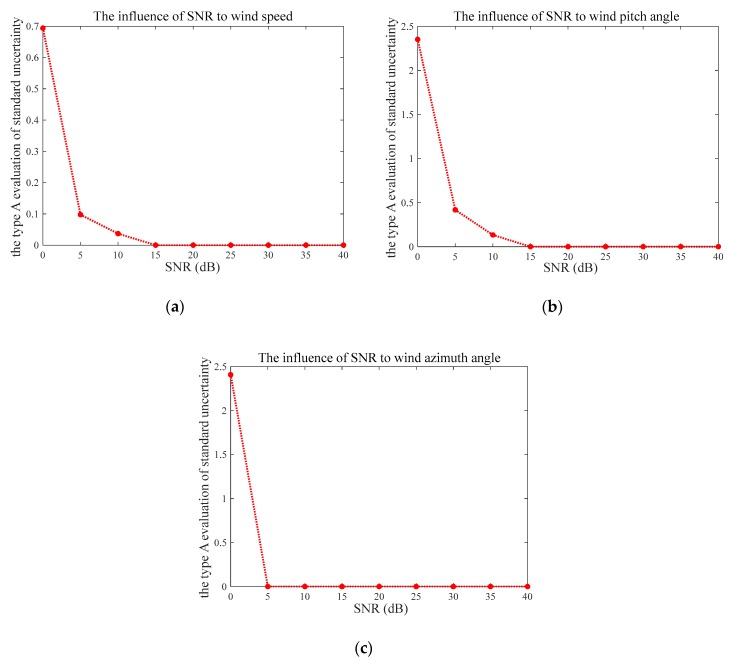
The type A evaluation of standard uncertainty of the results under different SNRs: (**a**) Measurement uncertainty of the wind speed, (**b**) measurement uncertainty of the wind pitch angle, and (**c**) measurement uncertainty of the wind azimuth angle.

To evaluate the stability of the proposed method, we performed 100 independent simulations with SNR ranging from 0 to 40 dB. The measurement deviation was quantitatively analyzed using the type A evaluation of standard uncertainty in Figure 4. As can be seen in Figure 4, the measurement uncertainty decreases with higher SNR. Specifically, the measurement uncertainty of wind speed V is 0.69 in the case of SNR at 0 dB, which sharply decreases to 0.09 at SNR of 5 dB, then drops to zero when the SNR is higher than or equal to 15 dB. The measurement uncertainty of pitch angle β shows a similar pattern, which begins at 2.35 at SNR = 0 dB, then falls to 0.42 at SNR = 5 dB and reaches zero at SNR ≥ 15 dB. However, the trend of the measurement uncertainty of wind azimuth angle θ is slightly different from those of the wind speed and pitch angle. The biggest uncertainty value of it is 2.41 when SNR = 0 dB, which then steeply falls to and remains at zero, since SNR is over 5 dB.

**Figure 5:**
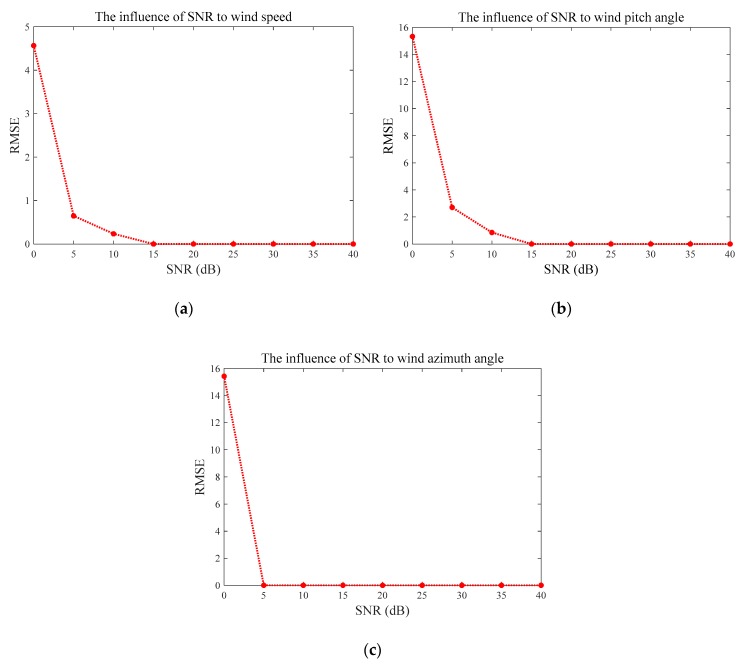
Results of Root Mean Square Error (RMSE) under different SNRs: (**a**) RMSE of the wind speed, (**b**) RMSE of the wind pitch angle, and (**c**) RMSE of the wind azimuth angle.

**Figure 6:**
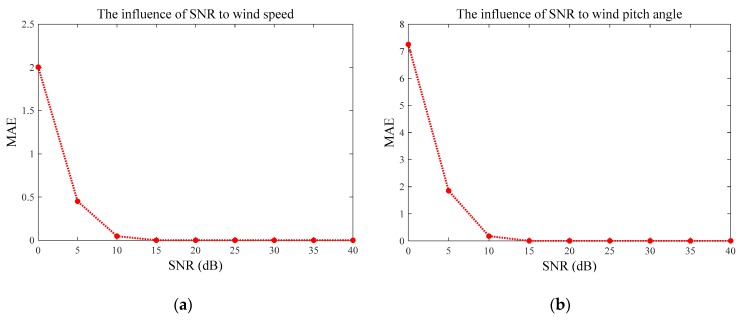

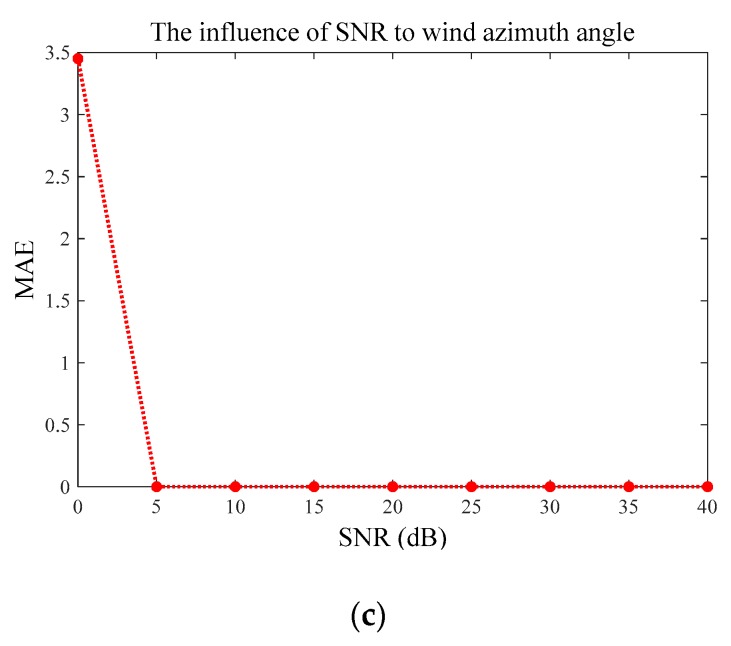
Results of mean absolute error (MAE) under different SNRs: (**a**) MAE of the wind speed, (**b**) MAE of the wind pitch angle, and (**c**) MAE of the wind azimuth angle.

The estimation errors are measured by Root Mean Square Error (RMSE) and Mean Absolute Error (MAE) in Figure 5 and Figure 6, respectively. Both of these figures illustrate quite consistent and similar patterns. The average errors of the wind speed V and pitch angle β converge to zero when SNR ≥ 15 dB, and these trends are consistent with those of the measurement uncertainty indicated by Figure 4a,b. Similarly, the RMSE and MAE of wind azimuth angle θ achieved zero, as SNR at 5 dB, whose variation under different SNRs is in line with what is shown in Figure 4c. Therefore, we can conclude that the proposed method is more reliable and more accurate with higher SNR, which is greater than 15 dB, to be precise.

### 3.2. Comparison with State-Of-The-Art Method

**Figure 7:**
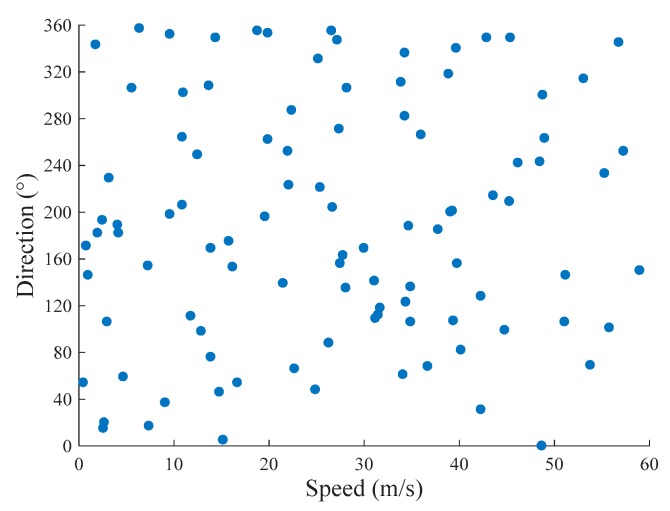
One-hundred groups of simulated wind speed and direction data.

The measurements of natural wind based on the MUSIC algorithm are widely performed in the 2D space. One of the representative methods for wind speed and direction measurement in the 2D space (WSDM2D) was proposed in [35], based on an arc ultrasonic sensor array. To compare the proposed method with WSDM2D, we set the pitch angle β to 90° to simulate the wind on the horizontal plane. The wind estimation problem was thus relegated to the 2D space in this case. We randomly simulated 100 groups of wind speed and direction to perform our estimates, where the former was uniformly distributed within 0–60 m/s, and the latter was uniformly distributed within 0 ∼ 360°. Figure 7 illustrates this distribution of the wind speed and direction data.

Monte Carlo simulations of 100 runs were carried out to compare the two methods with SNR ranging from 0 dB to 40 dB. Similar to the results in Section 3.1, the errors in the wind estimates of both the proposed method and WSDM2D converged to zero when SNR ≥ 15 dB. To perform a comprehensive comparison of the accuracy and anti-noise performance of the proposed method with WSDM2D, we calculated RMSE and MAE of speed and direction at SNR varying from 0 to 20 dB, the results of which are presented in the Table 1. Three findings can be deduced from Table 1:

1. The trends of RMSEs and MAEs are consistent, which all decrease with higher SNR.
2. Accordingly, the biggest RMSE and MAE of wind speed and direction occur at SNR of 0 dB. The biggest wind speed RMSE of the proposed method is slightly bigger than that of WSDM2D, while the biggest direction RMSE is smaller.
3. The speed and direction RMSEs of proposed method tend to zeros as SNR of 5 dB, outperforming the WSDM2D method, which converges to zero since SNR = 15 dB. Wind speed and direction MAEs of two methods have similar patterns with those of RMSEs.

**Figure 8:**
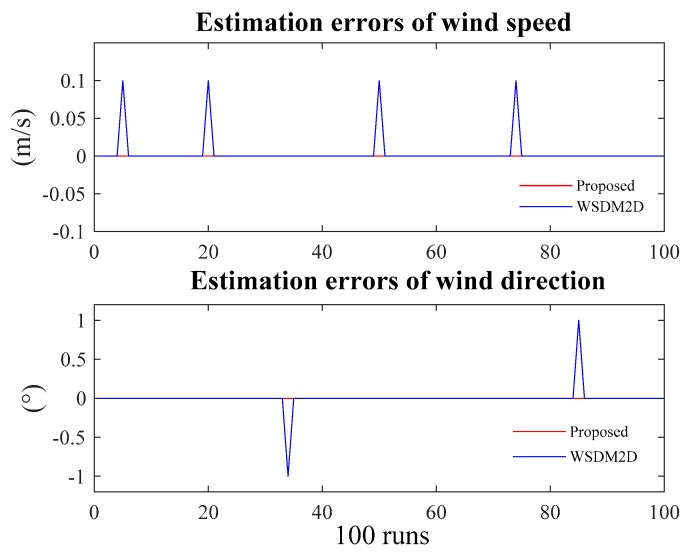
Estimation errors of wind speed and direction based on two methods at SNR of 5 dB.

Figure 8 depicts the errors in wind speed and direction of the two methods under the condition of SNR = 5 dB, where the red line represents the error of the proposed method and the blue line that of WSDM2D. It is obvious that the estimation errors of wind speed and direction are consistent and equal to zeros for the proposed method, whereas those of WSDM2D fluctuate.

In conclusion, the proposed method is more accurate and has better anti-noise performance than WSDM2D in wind speed and direction measurements, even in the 2D case.

## 4. Conclusions

We propose to measure the natural wind in the 3D space using an ultrasonic sensor array in the context of fluctuations in wind speed and production. More precisely, by building a novel semi-conical ultrasonic sensor array and using the MUSIC algorithm, we measure the wind speed V, azimuth angle θ, and pitch angle β simultaneously and accurately. The measurement deviation is quantified by the type A evaluation of standard uncertainty, and the estimation errors are evaluated using RMSE and MAE, respectively. The measurement errors of wind direction are within 1º, and those of wind speed within 0.1 m/s when the SNR is greater than 10 dB. Simulations show that the proposed method has better performance and higher accuracy when the SNR is greater than 15 dB, where errors are nearly zero. The measurement uncertainty and the estimation error illustrate similar patterns under the influence of varying SNRs, which further proves the consistency of the proposed method. Furthermore, we compared the accuracy of the proposed method with the state-of-the-art method in the 2D space. Monte Carlo simulations show that our method has higher accuracy and stronger noise immunity. Note that the structure of the sensor array is not limited to the one proposed in this study, and the accuracy of the measurement has room for improvement. To further verify the validity of the theory, we will perform practical experiments in the next step.

## Figures and Tables

**Table 1:** Comparison with WSDM2D.

SNR (dB)	RMSE				MAE			
	Speed		Direction		Speed		Direction	
	Proposed	WSD M2D	Proposed	WSDM2D	Proposed	WSDM2D	Proposed	WSDM2D
0	6.023	3.150	4.970	13.506	0.824	0.337	0.820	1.420
5	0	0.020	0	0.141	0	0.004	0	0.020
10	0	0.010	0	0.100	0	0.001	0	0.010
15	0	0	0	0	0	0	0	0
20	0	0	0	0	0	0	0	0
